# Vitamin B12 Deficiency Exhibiting as Pancytopenia: A Diagnostic Conundrum

**DOI:** 10.7759/cureus.87709

**Published:** 2025-07-11

**Authors:** Erica Lama, Susmita Sharma

**Affiliations:** 1 Internal Medicine, Manipal College of Medical Sciences, Pokhara, NPL; 2 Medical Oncology, Nepal Mediciti Hospital, Lalitpur, NPL

**Keywords:** anemia, cobalamin, diagnostic challenge, pancytopenia, vitamin b12 deficiency

## Abstract

Pancytopenia is caused by impaired production and peripheral destruction of blood cells, leading to decreased levels of red blood cells (RBCs), white blood cells (WBCs), and platelets. The etiologies can be classified as reversible and irreversible causes. One of the reversible causes is Vitamin B12 deficiency. Vitamin B12 deficiency can cause various types of manifestations, including hematologic as well as neurological symptoms, and sometimes mimicking serious hematologic conditions. A 46-year-old male presented with generalized body weakness, easy fatigability, and occasional nausea with vomiting for two months. His vital signs were stable, and on physical examination, icterus was present. The patient presented with anemia accompanied by leukopenia and thrombocytopenia on complete blood count, suggesting pancytopenia. Red blood cell indices showed macrocytosis, and the peripheral smear revealed teardrop cells along with normocytic normochromic cells, hypersegmented neutrophils, and few macroovalocytes and polychromatophils. Liver function tests showed elevated transaminases and indirect hyperbilirubinemia. A markedly elevated lactate dehydrogenase (LDH) level and low serum Vitamin B12 supported a diagnosis of megaloblastic anemia with associated hemolysis. During the hospital stay, two pints of packed red blood cells were transfused to correct anemia, and considering Vitamin B12 deficiency as the cause of pancytopenia, Vitamin B12 supplementation was started. At the time of discharge, the patient was symptomatically improving and hemodynamically stable. The patient was prescribed ursodeoxycholic acid, thiamine, and Vitamin B12 supplement. On follow-up, the patient was symptomatically better, and improvement was observed in lab findings as well. In brief, this case study showcases the vague clinical presentation of Vitamin B12 deficiency, making its diagnosis challenging. Therefore, Vitamin B12 deficiency should be considered as an important differential in cases with pancytopenia, which aids in its prompt treatment and eliminates unnecessary procedures.

## Introduction

Pancytopenia is a hematologic disorder caused by impaired production and peripheral destruction of blood cells, leading to decreased levels of red blood cells (RBCs), white blood cells (WBCs), and platelets. The etiologies can be classified as reversible and irreversible causes. Reversible causes include viral infections, such as Epstein-Barr virus or Parvovirus B19, chronic alcohol use disorder, deficiencies in Vitamin B12, folate, iron, and certain medications. As it can be caused due to various factors, thorough assessment should be done to find out the underlying cause [1,2]. 

Vitamin B12, also known as cobalamin, is a water-soluble vitamin. Its sources are dairy products, eggs, and red meat. It is mainly absorbed in the terminal ileum with the aid of intrinsic factor, a glycoprotein produced by parietal cells of stomach. It is involved in DNA, fatty acids, and myelin synthesis. Due to the large hepatic stores and the low daily requirement of Vitamin B12, clinical manifestations of its deficiency often take several years to develop. The causes of Vitamin B12 deficiency are drugs that led to decreased absorption (i.e., metformin), defect in its release from food (i.e., proton pump inhibitors, H2 receptor antagonists), and malabsorption, like in pernicious anemia, chronic pancreatic insufficiency, malnutrition, post-gastrectomy, chronic liver disease and atrophic gastritis. Vitamin B12 deficiency can cause various types of manifestations, including hematological, such as pancytopenia, neurological, such as peripheral neuropathy, and sometimes mimicking serious conditions like leukemia, thrombotic thrombocytopenic purpura (TTP), or myelodysplastic syndrome. It is also found that there is a higher prevalence of Vitamin B12 deficiency in elderly population due to decreased intestinal absorption of Vitamin B12. Measurement of plasma cobalamin is the gold standard diagnostic test for Vitamin B12 deficiency [1-5]. 

## Case presentation

A 46-year-old male, ex-tobacco user, with history of hepatitis B infection 25 years ago, for which he was treated with entecavir but lost to follow up after that, hypothyroidism under medication, and cholecystectomy, presented with generalized body weakness, easy fatiguability, and occasional nausea with vomiting for two months. He reported no recent exposure to ill individuals, travel, bleeding, blood in vomitus, black stool, shortness of breath, unintentional weight loss, alcohol consumption, and illicit drug use. 

The patient’s vital signs were stable: blood pressure was 130/80 mmHg, heart rate was 84 bpm, respiratory rate was 18 breaths/min, temperature was 98.1°F, and oxygen saturation rate was 98% on room air. On physical examination, icterus was present. On systemic examination, findings were normal. 

On initial blood analysis, HBsAg was reactive, whereas hepatitis C virus antibody test (HCV Ab), anti-hepatitis A virus (HAV) IgG, and anti-HAV IgM were non-reactive. On complete blood count (CBC), total leukocyte count was 3300 cells/µL, red blood cells were 1.79 million/µL, hemoglobin was 6.9 gms%, mean corpuscular volume (MCV) was 121.2 fl, platelet count was 78,000 cells/µL, and reticulocyte count was 2%. Liver function test (LFT) revealed alanine aminotransferase (ALT/SGPT) of 107 U/L, aspartate aminotransferase (AST/SGOT) of 202 U/L, total bilirubin of 2.5 mg/dl, and unconjugated bilirubin of 1.9 mg/dl. Lactate dehydrogenase was 4438 U/L, Vitamin B12 was 159 pg/ml, antinuclear antibody (ANA) was 0.74 AU/ml, iron was 255 µg/dl, iron saturation % was 74.78%, ferritin was 373 ng/ml, and C-reactive protein was 22.6 mg/L. On peripheral blood smear, teardrop cells, normocytic normochromic cells with hypersegmented neutrophils, and few macroovalocytes and polychromatophils were observed, and stool sample was negative for occult blood (Tables 1, 2). 

**Table 1:** Baseline investigation results with normal range. HBsAg: hepatitis B surface antigen; HCV Ab: hepatitis C virus antibody test; HAV: hepatitis A virus; HBV: hepatitis B virus.

Investigations	Result	Normal range
HBsAg	Reactive	
HCV Ab	Non-reactive	
Anti-HAV IgG	Non-reactive	
Anti-HAV IgM	Non-reactive	
HBeAg	Non-reactive	
HBV DNA titer (IU/mL)	<2000	Undetectable: <20; low viral load: 20–2000; intermediate viral load: 2000–20,000; high viral load: >20,000; active replication: >1 million
Lactate dehydrogenase (LDH) (U/L)	4438	45-90
Vitamin B12 (pg/ml)	159	300-950
Antinuclear antibodies (ANA) (AU/ml)	Negative (0.74)	Negative: <20; equivocal: 20–39; positive: ≥40
Iron (µg/dl)	255	50-170
Iron saturation (%)	74.78	15-50
Ferritin (ng/ml)	373	Male: 15-200; female: 12-150
C-reactive protein (mg/L)	22.6	<10

**Table 2:** Hematological response to Vitamin B12 supplementation.

Investigations	Day 1	Day 2	Day 10	Normal range
Total leucocyte count (cells/µL)	3300	4000	4240	4500-11,000
Red blood cells (million/µL)	1.79	2.39	2.98	Male: 4.3-5.9; Female: 3.5-5.5
Hemoglobin (gms%)	6.9	8.7	9.9	Male: 13.5-17.5; Female: 12-16
Mean corpuscular volume (MCV) (fl)	121.2	112.7	108.8	80-100
Platelet (cells/µL)	78,000	95,000	1,40,000	150,000-400,000
Alanine aminotransferase (ALT/SGPT) (U/L)	107		80	8-40
Aspartate aminotransferase (AST/SGOT) (U/L)	202		120	8-40
Total bilirubin (mg/dl)	2.5		1.9	0.1-1
Unconjugated bilirubin(mg/dl)	1.9		1.8	0.2-0.8

**Figure 1:**
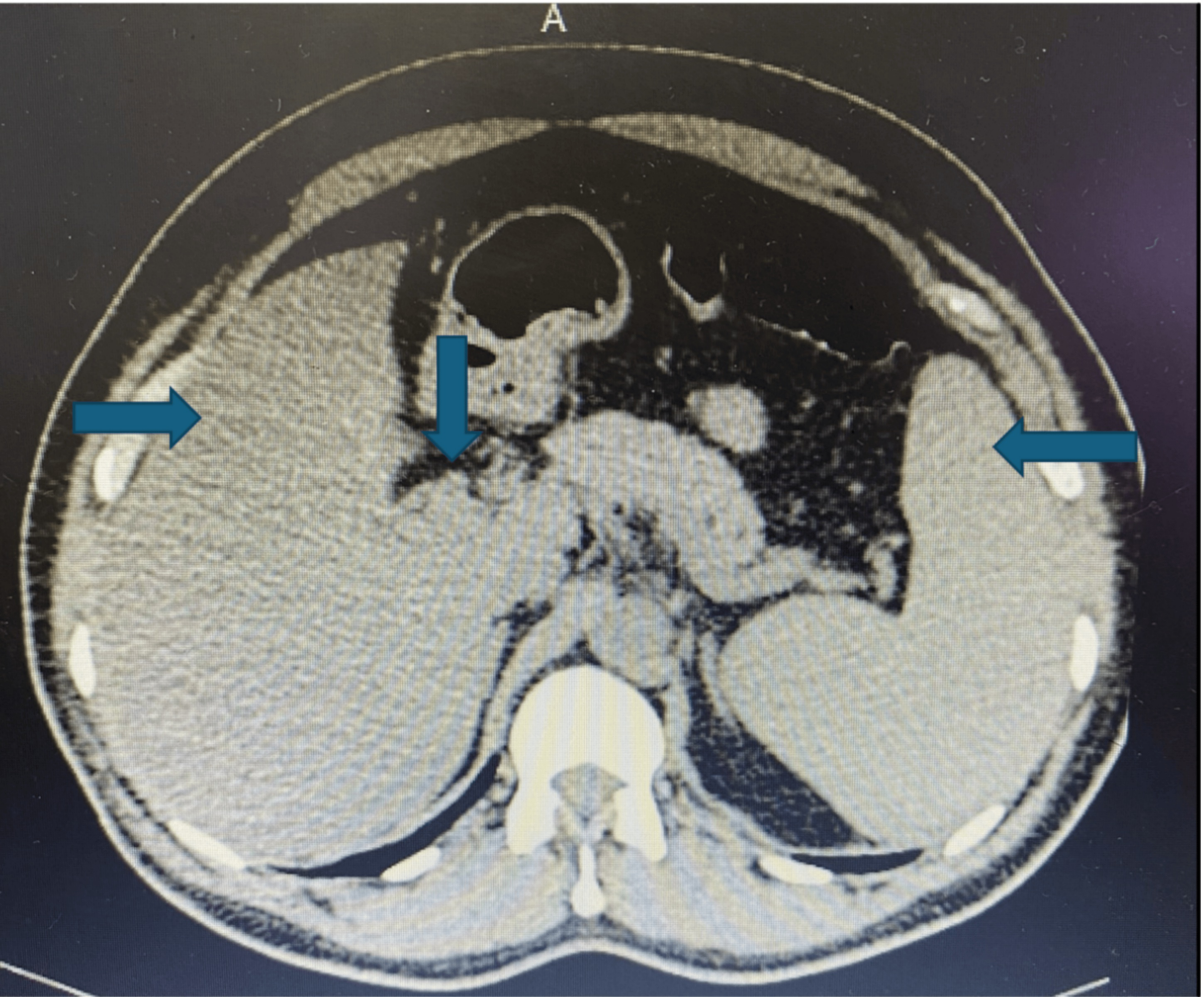
Computed tomography scan of the whole abdomen showing diffuse fatty liver, mild splenomegaly with dilated portal vein.

**Figure 2:**
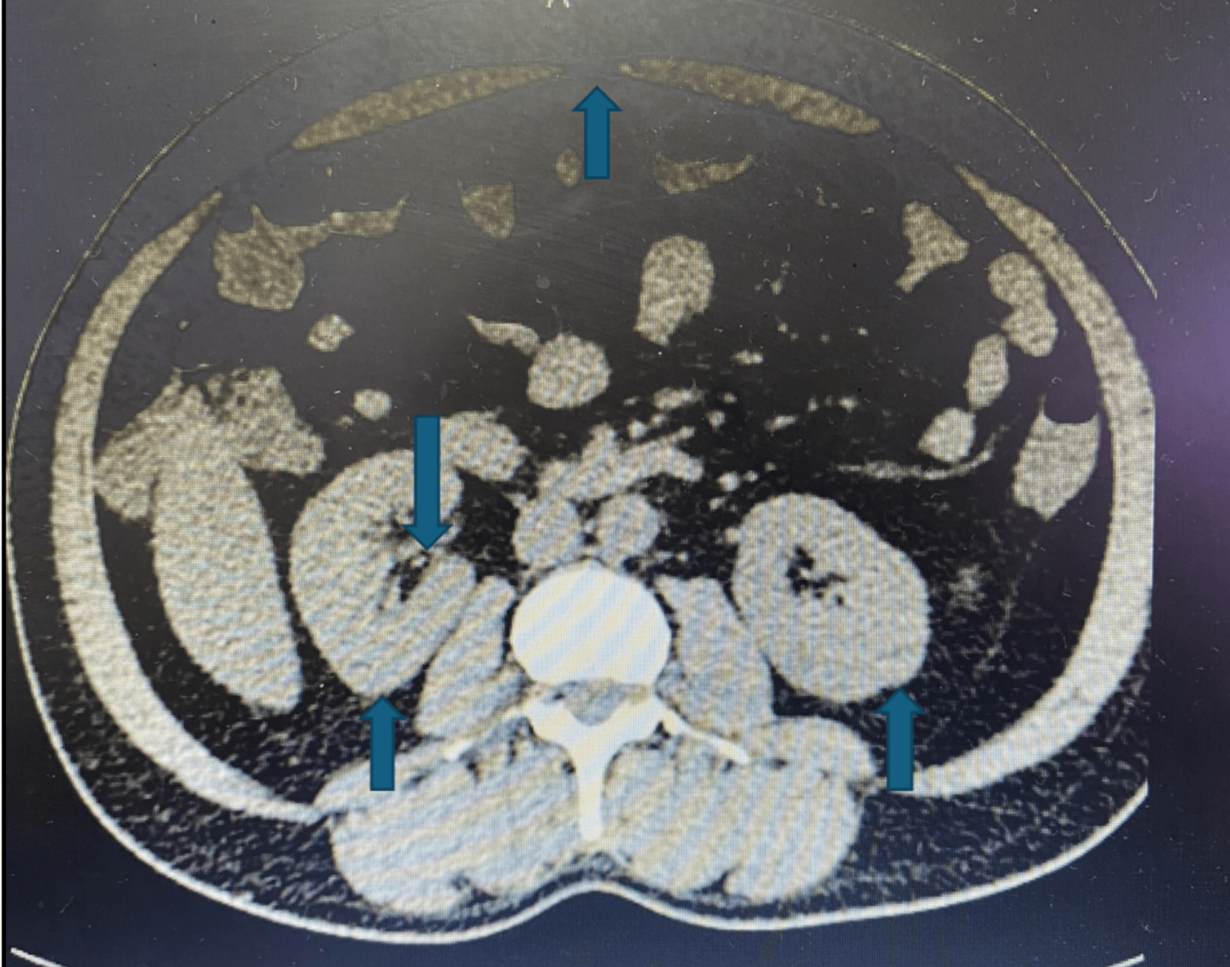
Computed tomography of the whole abdomen showing tiny right nephrolithiasis in the lower pole of the calyx, bilateral simple renal cortical cysts-Bosniak category 1, and small umbilical hernia.

Furthermore, Figures 1, 2 are the computed tomography scans of whole abdomen showing diffuse fatty liver, mild splenomegaly (13.93 cm) with dilated portal vein (14.1 mm), tiny right nephrolithiasis (2.3 × 2.2 mm) in lower pole of calyx, bilateral simple renal cortical cysts-Bosniak category 1 and small umbilical hernia (7.3 mm).

During the hospital stay, gastrointestinal medicine consultation was done for hepatitis B status, and further investigations were done and advised to follow up with reports. Two pints of packed red blood cells were transfused to correct anemia (post-transfusion Hb 9.3 gms%), and vitamin supplements were started. At the time of discharge, the patient was symptomatically improving and hemodynamically stable. The patient was prescribed an oral Vitamin B12 supplement, ursodeoxycholic acid, thiamine, and was advised to follow up in one week with CBC, LFT, HBeAg, and HBV DNA titer reports. 

On follow-up, the patient was symptomatically better and hemodynamically stable. According to the lab investigations, HBeAg was negative, and the HBV DNA titer was less than 2000 IU/mL. On LFT, ALT was 80 U/L, AST was 120 U/L, total bilirubin was 1.9 mg/dl, as well as unconjugated bilirubin was 1.8 mg/dl, and anti-viral was not started. Total leukocyte count was 4240 cells/µL, red blood cells were 2.98 million/µL, hemoglobin was 9.9 gms%, mean corpuscular volume (MCV) was 108.8 fl, and platelet count was 1,40,000 cells/µL. 

Given the patient's history and findings of chronic hepatitis B and elevated liver enzymes, hepatic dysfunction has contributed to the Vitamin B12 deficiency. Considering that Vitamin B12 deficiency is the cause of pancytopenia, Vitamin B12 supplementation was continued. 

## Discussion

This discussion will simplify and aid clinicians to identify the various causes of pancytopenia, specifically Vitamin B12 deficiency, considering its wide range of manifestations and manage it effectively. 

**Figure 3:**
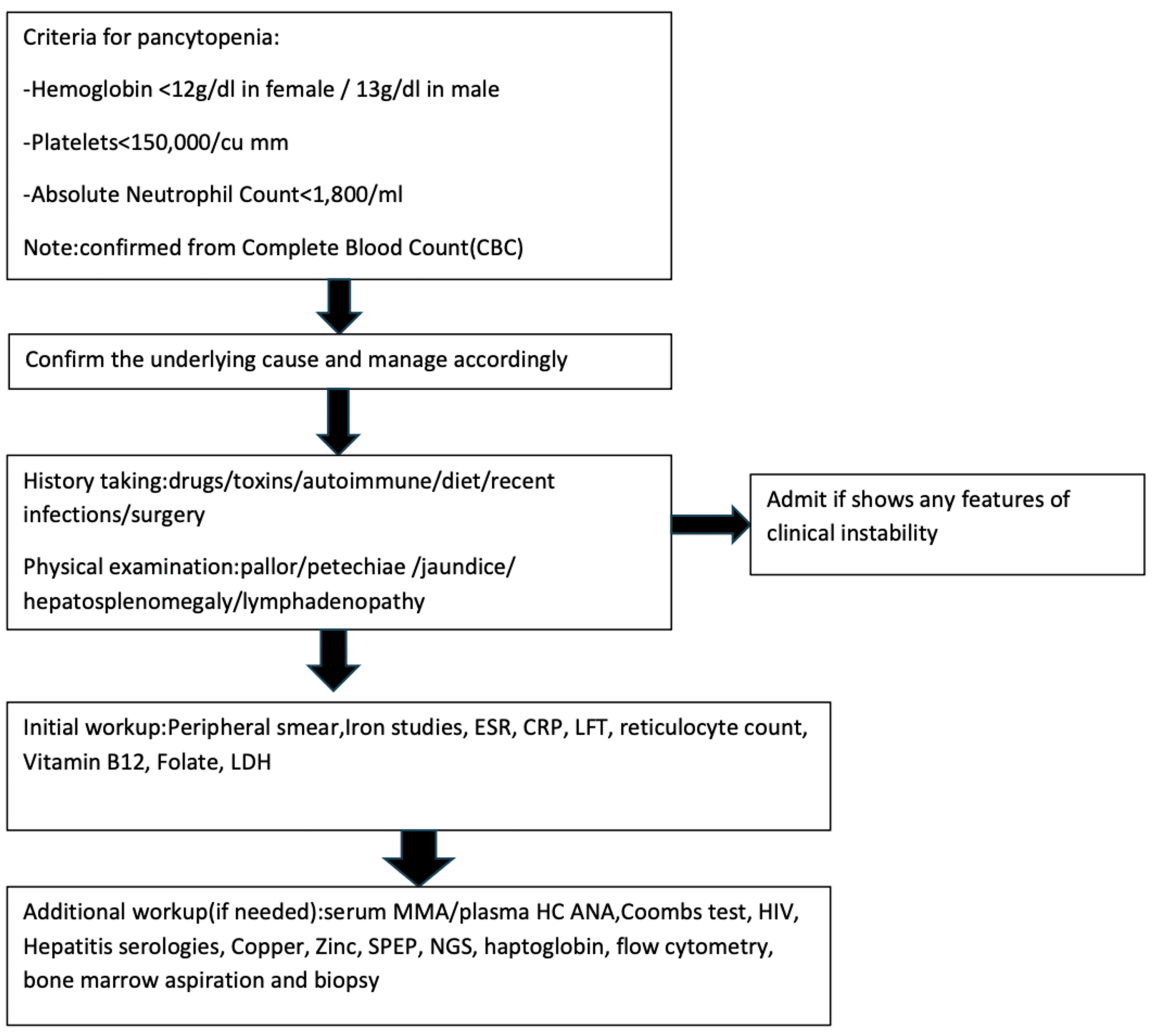
Workup algorithm for pancytopenia. ESR: erythrocyte sedimentation rate; CRP: C-reactive protein; LFT: liver function test; LDH: lactate dehydrogenase; MMA: methylmalonic acid; HC ANA: antinuclear antibodies with high concentration; SPEP: serum protein electrophoresis.

Pancytopenia can occur due to various etiologies and is often associated with other conditions. For prompt diagnosis and treatment, as shown in Figure 3, a thorough history and physical examination should be carried out, followed by appropriate management [1,6]. 

In our case scenario of pancytopenia with increased MCV, normal reticulocyte count, and teardrop cells, normocytic normochromic cells with hypersegmented neutrophils, few macroovalocytes and polychromatophils on peripheral smear ruled out aplastic anemia, iron deficiency anemia, and myelodysplastic syndrome. Negative ANA, normal body temperature, and absence of hepatomegaly ruled out an autoimmune cause. Increased LDH level with a low serum Vitamin B12 level indicates Vitamin B12 deficiency. Given the patient’s history and findings of chronic hepatitis B and elevated liver enzymes, hepatic dysfunction has contributed to the Vitamin B12 deficiency [1,7]. Therefore, the values of Vitamin B12 should be known for accurate interpretation, as given in Table 3. 

**Table 3:** Serum Vitamin B12 levels and their interpretation.

Serum level (pg/mL)	Interpretation
>300	Normal
200-300	Borderline
<200	Deficient

The clinical features seen in cobalamin-deficient individuals are given in Table 4. Regarding the treatment, administration of Vitamin B12 is the mainstay of treatment; however, the route of administration depends on the severity of the symptoms [8-13].

**Table 4:** Clinical features of Vitamin B12 deficiency.

System involved	Clinical manifestations
Neuropsychiatric manifestations	Headache, erectile dysfunction, spinal degeneration, peripheral neuropathy, epileptiform symptoms in <20 years old individuals and psychiatric, namely, depression, mania, Alzheimer’s, and delirium in >70 years old individuals
Oral manifestations	Hunter glossitis
Hematologic manifestations	Pancytopenia, decreased hemoglobin levels, hyper-segmented neutrophils, and increased mean corpuscular volume

In severe cases, the parenteral route is preferred; however, there are some contraindications to it, specifically in individuals who use anticoagulants. The common treatment given is injection of hydroxocobalamin 1 mg via the intramuscular route on alternate days for 14 days, and then weekly injection for one to two months, but cyanocobalamin is used in the United States. Regarding oral supplements, 1-2 mg of cyanocobalamin can be taken on a daily basis. In cases with severe neurological manifestations, the treatment is continued until no further relief is seen clinically, after which two injections are given per month. Since biomarkers often return to normal levels before clinical improvement, and conversely, in cases of relapse, they should not be solely relied upon for assessment and titrating the treatment [7,8,12-17].

## Conclusions

This case illustrates the importance to consider reversible and treatable causes of pancytopenia, such as Vitamin B12 deficiency. The overlapping hematologic features with serious hematological disorders, including myelodysplastic syndrome and aplastic anemia, posed a diagnostic challenge. However, careful interpretation of peripheral smear findings and targeted investigations helped avoid unnecessary invasive procedures. Clinicians should maintain a high index of suspicion for nutritional deficiencies in similar presentations, especially when supported by subtle morphological clues. Early identification and management can significantly improve outcomes and prevent misdiagnosis in resource-limited or complex clinical settings.
